# Soil phosphorous is the primary factor determining species-specific plant growth depending on soil acidity in island ecosystems with severe erosion

**DOI:** 10.1038/s41598-023-38934-9

**Published:** 2023-07-27

**Authors:** Kenji Hata, Syuntaro Hiradate, Naoki Kachi

**Affiliations:** 1 College of Commerce, Nihon University, 5-2-1 Kinuta, Setagaya-ku, Tokyo, 157-8570 Japan; 2 Department of Biological Sciences, Graduate School of Science, Tokyo Metropolitan University, 1-1 Minami-Osawa, Hachioji, Tokyo 192-0397, Japan; 3 Department of Agro-Environmental Sciences, Faculty of Agriculture, Kyushu University, 744 Moto-oka, Nishi-Ku, Fukuoka, 819-0395, Japan

**Keywords:** Restoration ecology, Invasive species, Environmental impact

## Abstract

Disturbances caused by invasive ungulates alter soil environments markedly and can prevent ecosystem recovery even after eradication of the ungulates. On oceanic islands, overgrazing and trampling by feral goats has caused vegetation degradation and soil erosion, which can alter soil chemistry. To understand the effects of the changes on plant performance, we conducted a laboratory experiment to assess herbaceous species growth under various soil conditions with phosphorous, nutrients, and acidity. Subsoil was collected from Nakodo-jima in the northwest Pacific. Six herbaceous species dominating the island were grown in soils with three levels of added CaCO_3_ and P_2_O_5_ and two levels of added KNO_3_. After 4 weeks of growth, the total dry plant weight was significantly lower with no added P_2_O_5_, regardless of the addition of KNO_3_. Three species weighed more under P_2_O_5_ and KNO_3_ addition in high-pH soil, whereas the remaining three weighed less. Our results indicated that herbaceous species growth is limited primarily by phosphorous availability; the limitation is dependent on soil pH, and the trend of dependency differs among species. This implies that ecosystems with extreme disturbances cannot recover without improving the soil chemistry.

## Introduction

Ongoing climate change and anthropogenic impacts are degrading ecosystems in many biomes globally. Natural ecosystems can recover to the original state after being disturbed by natural and anthropogenic events; this is called “resilience”^1^. However, if the disturbances exceed a threshold of resilience, the original state cannot be recovered^2^. The disturbances that cause irreversible changes are called “extreme events”. To promote the recovery of ecosystems that have undergone extreme modification, and to prevent extreme degradation, it is essential to understand the mechanisms of ecosystem resilience, as well as the environmental and anthropogenic factors that limit recovery.

The ecosystems of many oceanic islands are vulnerable to disturbances by invasive species^3^. Invasive ungulates have disturbed many oceanic island ecosystems^3–5^, which can cause terrestrial ecosystems to shift irreversibly. For example, overgrazing and trampling by feral goats, pigs, and sheep naturalized on islands have severely damaged the vegetation and soil environments on many island ecosystems^3,6,7^. As well as the direct effects of herbivory and digging^8,9^, overgrazing and trampling indirectly lead to soil erosion via vegetation degradation^8,10–12^. Conversely, in some instances, grazing and trampling by wildlife affect the plant cover and species composition but not the ecosystem functions^13^, suggesting that the effects of invasive ungulates are highly context dependent.

On Nakodo-jima (27°37′–27°38′N, 142°10′–142°11′E), a northwestern Pacific Ocean island, feral goats have inflicted significant harm on the ecosystem (refer to supplementary Fig. S1, online). These goats became naturalized after 1945, and their subsequent overgrazing and trampling for several decades led to serious vegetation degradation and soil erosion^14^. Although the island’s goat population was eradicated between 1997 and 1999^15^, the vegetation has yet to recover and soil erosion persists in certain areas^16^. This implies that the ecosystems experienced extreme disturbances surpassing their resilience threshold. It is likely that these ecosystems cannot recover without additional human intervention, even after goat eradication. To preserve and restore these ecosystems, understanding the processes and mechanisms behind the loss of ecosystem functions is crucial^17^.

The lack of ecosystem recovery after goat eradication is related to changes in soil chemistry with vegetation loss and soil erosion. Vegetation loss leads to exposed surface soil, promoting erosion that later alters the chemistry of the soil^18^. Loss of surface soil exposes the deeper subsoil layer, which is typically nutrient poor and highly acidic^19^. Hata et al. suggested that bare ground exposed by goat disturbance limits the primary production of herbaceous vegetation due to increased soil acidity^20^.

These studies suggested that changes in soil chemistry due to soil erosion limit plant performance, although this hypothesis was based on correlations in field surveys and has not been tested experimentally. To evaluate this hypothesis, it is necessary to quantify the effects of multiple soil chemical factors on plant growth because limitations to plant growth resulting from the chemistry of soil are the product of not only singular effects but also interactive effects among multiple chemical stressors. For example, soil pH and available phosphorus (P) interact such that the amount of available P is low in soils with a low pH, because labile P in soil combines with Fe^3+^ and Al^3+^, which are unavailable forms for plants^21^. Furthermore, available P can be converted to unavailable forms via combination with calcium carbonate in neutral to subalkaline soils^22^. Soil pH-related differences in nutrient availability originate from various P retention mechanisms in soils, which are influenced by the balances of non-labile, labile and solution P as well as their dynamics^23,24^. Thus, the extent to which P limits plant growth is dependent on soil pH; the extent of dependency can differ among species and soil environments.

To evaluate the impact of extreme changes in soil chemistry related to soil erosion on plant growth, we conducted laboratory experiments using subsoil collected from a layer exposed to severe soil erosion on Nakodo-jima. This soil has notably low nutrient levels and high acidity. Indeed, Bray-II P and pH (H_2_O) were significantly lower in soil of eroded areas than in less disturbed forests, with < 20 mg P_2_O_5_ 100 g^−1^ and < 5^19^. Additionally, sites with low vegetation coverage (< 13%) experienced 2 cm^2^ yr^−1^ soil loss and Bray-II P and exchangeable acidity values of < 20 mg P_2_O_5_ 100 g^−1^ and > 40 me kg^−1^ respectively^18^.

By manipulating soil nutrient levels and pH, we assessed the effects of essential soil nutrients and pH on the initial growth of herbaceous species, focusing specifically on available P. Phosphorus inputs in ecosystems primarily originate from rock weathering; hence, even minor losses in P cannot be easily replenished^25^. Isolated island ecosystems likely have even more limited P inputs^26,27^. Given this and the relationship between P availability and pH, we anticipated that the impact of soil erosion would be most pronounced on P availability compared to other nutrient elements such as nitrogen (N) and potassium (K). Indeed, Hiradate et al. suggested correlations among vegetation degradation caused by feral goats, available P, soil pH, and vegetation status in Nakodo-jima during goat invasion and subsequent eradication^19^.

We tested three predictions: first, the initial growth of plants will increase with the addition of available P and other nutrient elements; second, the magnitude of this growth increase will be dependent on soil pH; and third, the extent of dependency on nutrient elements and soil pH will differ among species. We compared the biomasses of six herbaceous species grown on the subsoil across various phosphorus pentoxide (P_2_O_5_), potassium nitrate (KNO_3_), and calcium carbonate (CaCO_3_) treatments; N and K are two other major essential macronutrients for plant growth. The KNO_3_ treatment was included to determine whether plant growth was limited by factors other than P, N and K. We hypothesized that plant growth is limited primally by available P. If plant growth under sufficient available P is limited secondarily by the availabilities of N and K, the addition of N and K should enhance plant growth. The six study species (three Poaceae and three Asteraceae, Table 1) are non-native species but comprise a large proportion of the herbaceous vegetation (i.e., dominant species) that was disturbed by goats and recovered after goat eradication in the Ogasawara (Bonin) Islands, including Nakodo-jima. Based on our results, we discuss the restoration of ecosystems whose functions have been lost as a result of severe disturbances following ungulate eradication.

**Table 1:** The six herbaceous species used in the laboratory experiments and experimental durations.

Species		Family	Experiment period
*Paspalum dilatatum*	PasDil	Poaceae	2016/01/11–2016/02/10
*Paspalum scrobiculatum*	PasScr	Poaceae	2016/04/12–2016/05/16
*Sporobolus diander*	SpoDia	Poaceae	2016/10/13–2016/11/11
*Bidens pilosa*	BidPil	Asteraceae	2016/08/01–2016/08/31
*Emilia sonchifolia*	EmiSon	Asteraceae	2016/08/15–2016/09/17
*Symphyotrichum subulatum*	SymSub	Asteraceae	2016/09/08–2016/10/11

Life forms, phenologies, and seed morphologies of the species are presented in Supplementary Table S1.

## Results

Over the 4-week experiment, out of 162 seedlings per species (3 P_2_O_5_ levels × 3 KNO_3_ levels × 2 CaCO_3_ levels × 9 replicates), five *Paspalum dilatatum*, one *Sporobolus diander*, six *Bidens pilosa*, and nine *Emilia sonchifolia* seedlings died. All of the *Paspalum scrobiculatum* and *Symphyotrichum subulatum* seedlings survived the entire study.

**Figure 1:**
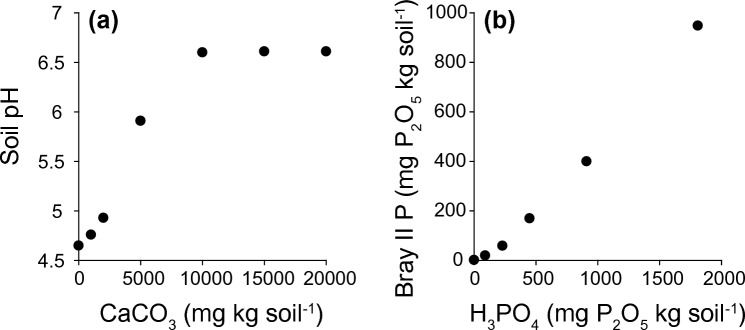
Relationships (**a**) between soil pH and the amount of CaCO_3_ added, and (**b**) between soil-available phosphorus (Bray-II P) and the amount of P_2_O_5_ added.

The ranges of total dry plant weights at the end of the experiment were 0.010–0.332 g pot^−1^ for *P*. *dilatatum*, 0.012–0.496 g pot^−1^ for *P*. *scrobiculatum*, 0.002–0.112 g pot^−1^ for *S*. *diander*, 0.025–0.377 g pot^−1^ for *B*. *pilosa*, 0.003–0.330 g pot^−1^ for *E*. *sonchifolia*, and 0.0008–0.082 g pot^−1^ for *S. subulatum*. In preliminary experiments, we confirmed that addition of CaCO_3_ increased soil pH (Fig. 1a), whereas addition of P_2_O_5_ increased the amount of available P in the soil (Fig. 1b).

**Figure 2:**
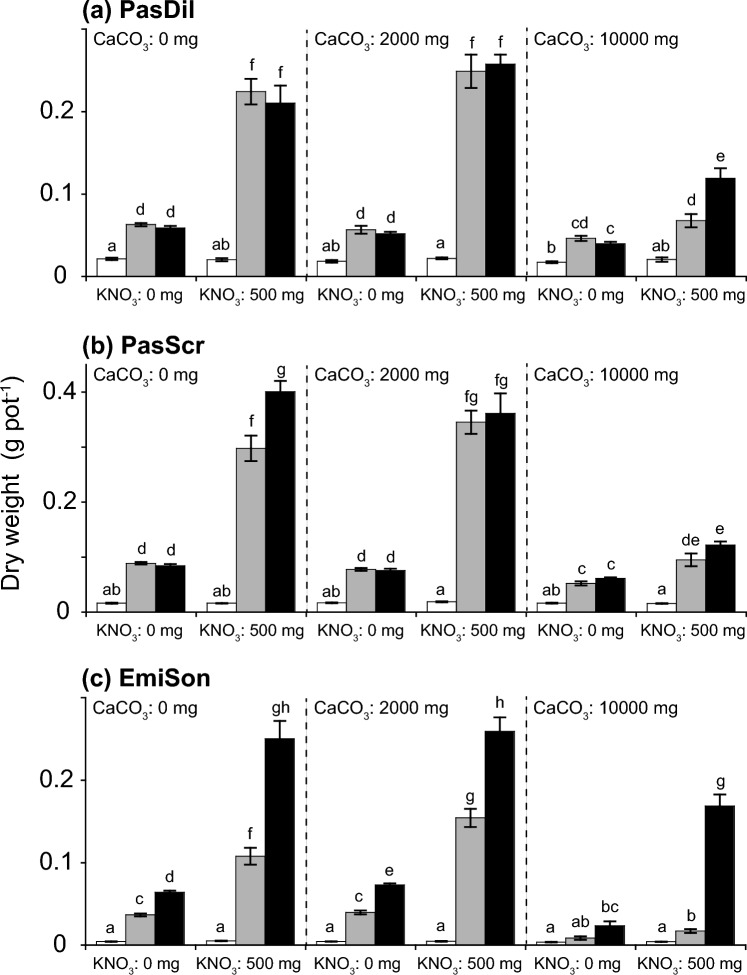

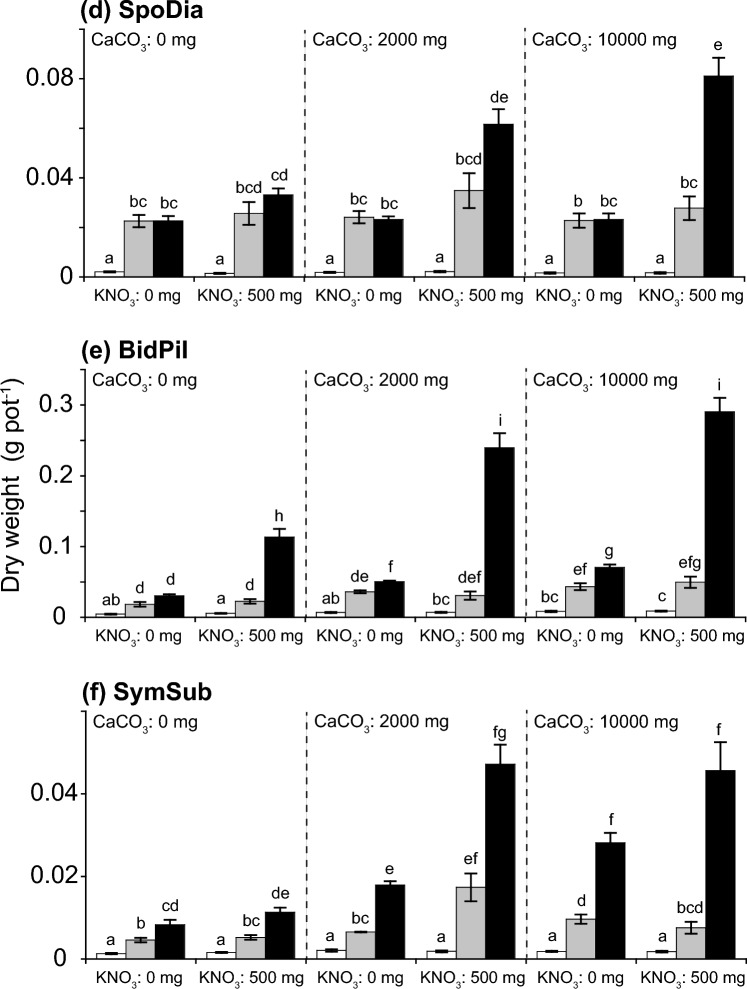
Total dry weights (g pot^−1^) of investigated species under 18 combinations of three treatments (3 × 3 × 2). White, gray, and black bars represent 0, 250, and 500 mg P_2_O_5_ kg soil^−1^ concentrations, respectively. Mean values (columns) and standard errors (bars) are shown. Letters indicate significant differences (*p* < 0.05) among treatments based on Holm’s multiple comparison method after generalized linear mixed model (GLMM) analyses. (**a**) PasDil, *Paspalum dilatatum*; (**b**) PasScr, *Paspalum scrobiculatum*; (**c**) EmiSon, *Emilia sonchifolia*; (**d**) SpoDia, *Sporobolus diander*; (**e**) BidPil, *Bidens pilosa*; (**f**) SymSub, *Symphyotrichum subulatum*.

For all the investigated species, the total dry weights were significantly affected by the addition of P_2_O_5_ (Table 2). Seedlings were significantly smaller in the treatments without P_2_O_5_ addition than in those with P_2_O_5_ addition, regardless of the addition of CaCO_3_ or KNO_3_ (Fig. 2). We found a significant interaction effect between P_2_O_5_ and KNO_3_ additions for all species (Table 2), indicating that the growth response to KNO_3_ addition was dependent on the addition of P_2_O_5_. The total dry weights increased with the addition of both KNO_3_ and P_2_O_5,_ but not with the addition of KNO_3_ alone (Fig. 2).

**Table 2:** Effects of treatments (CO_3_, P_2_O_5_, and KNO_3_ addition and their interactions) on total dry plant weight, as tested by generalized linear mixed models (GLMMs).

Explanatory variable	d.f	(a) PasDil		(b) PasScr		(c) EmiSon		(d) SpoDia		(e) BidPil		(f) SymSub	
		χ^2^		χ^2^		χ^2^		χ^2^		χ^2^		χ^2^	
CaCO_3_	2	157.9	***	382.6	***	292.1	***	9.9	**	158.1	***	172.5	***
P_2_O_5_	2	1848.9	***	5670.8	***	2481.4	***	2008.8	***	1969.8	***	1619.8	***
KNO_3_	1	577.1	***	953.2	***	321.3	***	34.6	***	109.0	***	32.3	***
CaCO_3_ × P_2_O_5_	4	62.8	***	163.4	***	125.5	***	10.3	*	8.5	n.s	59.1	***
CaCO_3_ × KNO_3_	2	42.0	***	113.1	***	0.4	n.s	12.5	**	0.9	n.s	19.9	***
P_2_O_5_ × KNO_3_	2	235.9	***	461.1	***	115.4	***	43.9	***	165.9	***	20.7	***
CaCO_3_ × P_2_O_5_ × KNO_3_	4	39.2	***	43.6	***	22.1	***	5.0	n.s	5.0	n.s	23.9	***

Degrees of freedom (d.f) and Chi-square values (χ^2^) are shown for explanatory variables. (a) PasDil, *Paspalum dilatatum*; (b) PasScr, *Paspalum scrobiculatum*; (c) EmiSon, *Emilia sonchifolia*; (d) SpoDia, *Sporobolus diander*; (e) BidPil, *Bidens pilosa*; (f) SymSub, *Symphyotrichum subulatum*.

*** *p* < 0.001, ** p < 0.01, * *p* < 0.05, n.s. = not significant.

The effect of CaCO_3_ addition on total dry seedling weights was dependent on P_2_O_5_ and KNO_3_ additions. There was evidence of a significant effect of CaCO_3_ addition for all species, as well as evidence of interactions between CaCO_3_ and P_2_O_5_ and between CaCO_3_ and KNO_3_ (Table 2). In agreement with these findings, the investigated species were roughly classified into two types according to their treatment responses. Type 1 included *P*. *dilatatum*, *P*. *scrobiculatum*, and *E*. *sonchifolia* and type 2 included *S*. *diander*, *B*. *pilosa*., and *S. subulatum*. For type 1 species, total dry seedling weight considerably increased with the addition of P_2_O_5_ and KNO_3_ when no or little of CaCO_3_ was added (Fig. 2a–c). For type 2 species, total dry weight substantially increased with the addition of P_2_O_5_ and KNO_3_ in the presence of high CaCO_3_ addition (Fig. 2d–f). Type 2 species were further classified into two types (Type 2a: *S*. *diander* and *B*. *pilosa*; Type 2b: *S. subulatum*) according to their treatment responses, especially to changes in the amounts of CaCO_3_. Compared with Type 2a (*S*. *diander* and *B*. *pilosa*), Type 2b (*S. subulatum*) appeared to be more affected by CaCO_3_.

## Discussion

### Limitation of plant growth on changing soil chemistry

Our results indicated that the initial growth of herbaceous species will be limited in soils whose chemistry has been affected by severe erosion (i.e., surface soil loss and exposed subsoil), in agreement with the relationship between herbaceous species and soil chemistry found in this study^18^. Surface soil loss due to vegetation degradation exposes the deeper subsoil below, which on Nakodo-jima is highly acidic and nutrient poor^19^. Few plants occur at these sites^18^, likely because of limited seed or propagule dispersal^28,29^ or poor plant growth and survival. Our results suggested that it is unlikely that herbaceous species can survive or thrive at sites that have experienced heavy soil erosion, even following goat eradication and assuming sufficient seed or propagule dispersal.

The initial growth and survival at heavily eroded sites are most likely related to soil deficiencies in nutrients, especially available P. The reduced biomass observed in seedlings that did not receive P_2_O_5_ additions, regardless of CaCO_3_ and KNO_3_ additions (Fig. 2), suggested that available P is the primary limiting factor for initial growth in the degraded soil systems on Nakodo-jima. Indeed, the amount of soil-available P recorded at eroded sites on Nakodo-jima [29.72 ± 9.85 mg P_2_O_5_ kg soil^−1^ (Bray-II P), Hata et al.^18^], would be insufficient for the establishment of many herbaceous species^30,31^. Evidence of P limitation was also shown in the above- and below-ground biomass allocations in seedlings. The shoot:root ratios of all species, excluding *B*. *pilosa*, tended to be lower in treatments without P_2_O_5_ addition (see Supplementary Fig. S2 online), indicating that a P deficiency may be related to an increase in root biomass in an effort to acquire P from the soil^32,33^. However, the significant interaction between the addition of P_2_O_5_ and KNO_3_ (Table 2) demonstrated that N and K can also have substantial effects on plant growth in denuded soils when P availability is adequate (Fig. 2). This may be explained by the interaction between available P and N in soils, as available N increases with the addition of P^34^.

Significant interactions between CaCO_3_ and P_2_O_5_ and between CaCO_3_ and KNO_3_ additions (Table 2) indicated that the effect on plant growth caused by the addition of N, K and P was dependent on soil pH, which was consistent with our second prediction. This dependency, in part, is explained by the relationship between soil nutrient availability and pH. Available P is typically low in low pH soils because labile P forms stable complexes with Fe^3+^ and Al^3+^, which are unavailable forms for plant uptake^21,35^. These unavailable forms of P dissolve and become available to plants as soil pH approaches neutral. This was exemplified in the response of type 2 species in our study, growth increased in *S*. *diander*, *S. subulatum*, and *B*. *pilosa* with nutrient addition when soil pH was relatively high (Fig. 2d–f). However, type 1 species displayed the opposite response (Fig. 2a–c), suggesting that the addition of P, N and K has minimal impact in neutral pH soil. This result may be associated with growth limitations caused by reduced supplies of heavy metals such as Fe, Zn, or Cu under mildly acid and neutral pH conditions^35,36^.

These varied responses to soil pH could be related to species-specific tolerances to soil acidity and/or limitations regarding available nutrients^37–39^. Besides the previously mentioned limitation of available P in low pH soil^21,35^, there are also evidences that available P is lower in near neutral pH soil^22,23^. The diverse responses to soil pH suggests that there are multiple dynamic mechanisms among P reactions (non-labile, labile and solution) in soil depending on pH. These mechanisms are related to soil environmental factors such as anion exchange, precipitation of Ca, Al, and Fe phosphates, and ligand exchange^24^. Taking these mechanisms into account, further investigation of plant responses to available nutrients, as well as plant uptake under various soil environments, will contribute to a better understanding of the factors limiting plant establishment in severely eroded soil environments.

### Conclusion and implications for the restoration of extremely altered ecosystems

We demonstrated that extremely altered soil chemistry, caused by severe soil erosion resulting from vegetation degradation induced by feral goats, limits the initial growth of herbaceous species. Therefore, disturbances that dramatically alter the soil environment (e.g., disturbance caused by invasive ungulates) may prevent the re-establishment of plants even after the stressor (i.e., feral goats) is removed. Furthermore, our experiment successfully quantified the interactive effects of multiple soil chemistries on plant performance and highlighted the importance of these effects. Indeed, some of the interactions were stronger than single effect (Table 2), suggesting that the dependency of soil pH on nutrient availability can influence the re-establishment of plants. The findings contribute to our understanding of the processes underlying ecosystem function loss and will aid predictions of the recovery of ecosystems with anthropogenically and naturally altered soil chemistry.

Limited or no natural recovery in heavily disturbed ecosystems following invasive species eradication suggests that the altered functional states of ecosystems changed by disturbances are irreversible. In the case of Nakodo-jima, ecosystem function has been lost in goat-disturbed soils. Given that the ecosystem is not resilient to this loss and therefore cannot return naturally to its pre-disturbance state (i.e., historical condition), Nakodo-jima is considered a “novel ecosystem”^40^. Management is therefore necessary to increase stability in this novel ecosystem^41^. Management actions that could stabilize or restore the soil environment and allow for plant re-establishment include soil control dams, fertilizer application, and/or soil amendments in eroded soils. Additionally, biochar can be an effective tool for the improvement of P availability in soil^42^, which could enhance plant growth via alteration of mycorrhizal activities^43^.

In the context of anthropogenic fertilization of soils with low nutrient levels and strong acidity, our results could help identify the fertilizer combinations that can affect the establishment of herbaceous species. For example, the addition of P, N, and K, which are usually included in commercial fertilizers, can promote the establishment of plant species like type 1 in this study (*P*. *dilatatum*, *P*. *scrobiculatum*, and *E*. *sonchifolia*), which have higher growth rates in soils with lower pH. Conversely, it is necessary to supply P, N, K, and CaCO_3_ for plant species like type 2 (*S*. *diander*, *S. subulatum*, and *B*. *pilosa*), which grow better in soils with higher pH.

As well as anthropogenic fertilization, soils in disturbed ecosystems on oceanic islands can be fertilized naturally by seabird nesting via the deposition of feces, eggshells, and carcasses^44–46^. Seabird nesting typically decreases in the presence of invasive mammals because of predation^47^ and trampling^7^, but often recovers following invasive species eradication^48,49^. Indeed, the populations of several dominant seabird species on Nakodo-jima have increased since goat eradication^50^, and higher soil nutrient concentrations were detected at sites with seabird nesting following eradication^16,20^. Seabird population recovery has been shown to alter soil pH as well as soil nutrients, although these effects are likely to be species-specific^51^. In addition, the species composition of seabird communities that recover after goat eradication can differ from those before eradication^52^. Thus, seabird recovery following invasive ungulates eradication cannot always promote plant growth by altering soil chemistry. A comprehensive understanding of species-specific growth responses to altered soil chemistry both before and after the introduction of invasive species will enable more effective and practical plant and ecosystem management and restoration practices.

The use of non-native herbaceous species instead of native ones was related to the observation that only few native herbaceous species were able to recover after goat eradication on the island. It is less likely that native herbaceous species can re-establish on the island after goat eradication because of the lack of seed and propagule supplies. A top priority for ecosystem restoration should be the protection of soil environments to prevent further loss of ecosystem functions of soil. Although the restoration of ecosystems degraded by invasive ungulates ideally should be conducted using native species that were present before degradation, this can be difficult in some situations, such as an island ecosystem with loss of ecosystem functions. In these situations, the first priority should be restoration of ecosystem functions (e.g., stabilization of soil environments in this study) regardless of native or non-native species rather than the biotic compositions of the original communities^53^. If non-native species are used for ecosystem restoration, the second priority should involve evaluating how the establishment of non-native species can alter the ecosystem functions and how the alteration can affect the subsequent establishment of native species.

## Methods

### Soil collection site

The soil samples used in the laboratory experiments were collected from a grassland area on Nakodo-jima (27°37′–27°38′N, 142°10′–142°11′E, 1.37 km^2^) in the Ogasawara (Bonin) Islands, a subtropical archipelago in the northwestern Pacific Ocean (Supplementary Fig. S1 online). The mean precipitation and annual temperature on Chichi-jima, the largest island, were 1305 mm and 23.3 °C, respectively, during 1995–2014 (Japan Meteorological Agency, Tokyo, Japan).

Many areas of Nakodo-jima were likely forested before the introduction of goats^14^. Goats became naturalized on Nakodo-jima by 1945^54^. They had access to most areas of the island and severely damaged the native vegetation through grazing and trampling^14,54^. Goat grazing on tree and shrub seedlings hindered native forest regeneration after the death of canopy trees^14^. This lack of regeneration led to a shift from forested to grassland areas. The grassland vegetation was roughly classified into two types; one dominated by *P. dilatatum* and *P. scrobiculatum*, and the others dominated by *Zoysia tenuifolia*^14^.

Grasslands dominated by *P. dilatatum* and *P. scrobiculatum* experienced continued grazing and trampling, resulting in grassland loss and surface soil exposure until the early 1990s^54^. Exposed surface soils were then subjected to erosion by wind and rain. Consequently, areas of forest and grassland decreased, while areas of bare ground increased. For instance, the proportion of forest area declined from 14.6% in 1968 to 8.6% in 1991^54^. This is in agreement with the re-survey of aerial photos between 1978 and 1991, which showed a decrease of forests and grasslands and an increase of bare ground areas^54,55^.

Feral goats were eradicated from the archipelago between 1997 and 1999^14^. After eradication, the natural recovery of herbaceous vegetation, which was dominated by non-native species rather than native species, was patchy (i.e., it occurred at some sites but not others)^55^. In 2012, many areas of bare soil still remained^56^.

### Soil collection method

To obtain soil with low nutrient levels and pH, a sample was collected from the subsoil layer (5–80 cm depth, B-layer) in an area with grassland cover on Nakodo-jima in July 2013. Soil was collected at one site only because our objective was to evaluate plant growth in response to nutrient addition and increased pH for soil with extremely low nutrient levels and low pH, rather than to evaluate relationships between plant growth and soil chemistry. Grassland had a total coverage of approximately 80% and it was mainly composed of *P. dilatatum* and *P. scrobiculatum* (Hata, K. personal observation). We collected approximately 300 kg raw-weighted soil from > 10 locations in one area within a ~ 5-m radius range and mixed the sample.

The collected soil was frozen at − 30 °C in a deep freezer for 48 h to reduce the effects of soil microbes. The sample was thoroughly mixed and sieved through a 5-mm mesh; visible plant residues in the soil sample were carefully removed using tweezers. Soil was air-dried within the Tokyo Metropolitan University Ogasawara Field Research Station in Chichi-jima between July 30 and November 14. The air temperature in the station ranged approximately from 25 to 35 °C. The air-dried soil was then stored in sealed polyethylene bags in an environment without direct sunlight until the experiments began (Table 1). The soil pH and available P of the sample were estimated as 4.56 ± 0.07 and 29.72 ± 9.85 mg P_2_O_5_ kg soil^−1^ (Bray II P) (mean ± standard error), respectively, and were determined according to the method of Hata et al.^18^. The sieved and air-dried soil had a very low total carbon level (0.84%, see Supplementary Table S2 online) such that the effects of soil microbes on soil mineralization would be small.

### Study species and collection of the seeds

The initial growth of six herbaceous species (*Paspalum dilatatum*, *Paspalum scrobiculatum*, *Sporobolus diander*, *Bidens pilosa*, *Emilia sonchifolia*, and *Symphyotrichum subulatum*) was evaluated in this study (Table 1). All six species are non-native but have been dominant herbaceous species in grasslands on Nakodo-jima since goat eradication, where native herbaceous species are not dominant^20^. These species are also dominant around soil sample collection sites. Seeds from each species were collected from grasslands around the sampling site and similar grasslands on Nakodo-jima in July 2015 and 2016. Seeds were stored in sealed containers with silica gel at the Plant Ecology Laboratory, Tokyo Metropolitan University, Hachioji, Japan (35°37′ N, 138°22′ E), until the experimental trials began.

### Laboratory experiments

Laboratory experiments were conducted in a temperature-controlled chamber (Koito-toron KG-50-HLA, Koito Manufacturing, Tokyo, Japan) at the Tokyo Metropolitan University. The chamber temperature was maintained at 25 °C during the day (16 h) and 18 °C at night (8 h), which is roughly similar to the field conditions on Nakodo-jima. The light intensity in the chamber was ~ 650 μmol m^−2^ s^−1^.

In a randomized block design, the experiment included 18 treatments derived from combinations of three concentrations of CaCO_3_ (0, 2,000, and 10,000 mg kg soil^−1^), three concentrations of P_2_O_5_ (0, 250, and 500 mg kg soil^−1^), and two concentrations of KNO_3_ (0 and 500 mg kg soil^−1^). We conducted nine replications (blocks) per treatment. The concentrations of P_2_O_5_ were determined based on vegetated soil value on Nakodo-jima (mean value; 202 mg kg soil^−1^, Hiradate et al.^19^). The concentration of KNO_3_ was approximately equivalent to 80 kg N ha^−1^, which falls within the standard fertilization range in cultivated areas in Japan^57^.

We added powdered CaCO_3_, a 10,000 ppm solution of H_3_PO_4_ (as P_2_O_5_), and powdered KNO_3_ to the air-dried soil and mixed them well in a polyethylene bag for each respective treatment. For the plant growth experiment, each pot contained air-dried soil equivalent to 40 g oven-dried soil. We mixed 162 soil samples (18 treatments × 9 replicates per treatment) and filled polypropylene pots (50 cc) for each of the six study species. The pots were placed in the chamber using a randomized block design.

We sowed more than 1000 seeds from each species on a 50 × 35 × 8 cm plastic tray filled with vermiculite and stored in the chamber until germination. The tray was watered without sterilization using a spray bottle every 1–2 days, ensuring that the vermiculite did not dry out. We transplanted cotyledon-stage seedlings into the treatment pots filled with soil and nutrients. If a planted seedling died within 1 week of transplanting, we replaced it with a new transplant. Pots were watered every 1–2 days, providing sufficient water and minimizing limitations on plant growth.

Four weeks after transplanting, planted seedlings were divided into shoot and root portions and dried at 70 °C for 72 h. Shoot and root dry weights were measured separately. The laboratory experiment was conducted from October 2015 to November 2016 (Table 1).

### Statistical analyses

All statistical analyses were performed using R ver. 4.1.2.^58^. Generalized linear mixed models (GLMMs) were applied using the *glmer* package, wherein the response variable was the total dry weights of plants, with a gamma distribution and a log link function. Each of the six species was analyzed separately. Seven factors (CaCO_3_, P_2_O_5_, and KNO_3_ concentrations and four interactions among them, see Table 2) were used as explanatory variables. Blocks (n = 9) were treated as a random effect. After the GLMMs, all possible pairwise combinations of treatments were investigated, again using treatments as the explanatory variables and blocks as a random effect. The resulting *p*-values were adjusted using Holm’s method^59^.

### Research involving plants

We obtained appropriate permission to collect the seeds of plants used in this study from the Ministry of Environment in Japan and the Department of National Forests in the Ogasawara Islands, Tokyo Metropolitan Government. We collected the seeds of plant species in compliance with relevant guidelines and legislation of the Ministry of Environment, Japan.

## Supplementary Information

Supplementary Information 1.

Supplementary Information 2.

Supplementary Information 3.

Supplementary Information 4.

## Data Availability

All data generated or analyzed in this article are included in supplementary information files as follows: (1) Hata_et_al_supplementary_fig_table_r3_20230721 (PDF file)*. (2) Hata_et_al_supplementary_dataset_fig_2_r1_20230309 (xlsx file). (3) Hata_et_al_supplementary_dataset_fig_3_tabel_2_r1_20230309 (xlsx file). (4) Hata_et_al_supplementary_dataset_fig_s2_r1_20230309 (xlsx file).
