# Supplementary material for: Soil phosphorous is the primary factor determining species-specific plant growth depending on soil acidity in island ecosystems with severe erosion
Source: Sci Rep. 2023 Jul 27;13:12163. doi: 10.1038/s41598-023-38934-9 (PMC10374522; doi:10.1038/s41598-023-38934-9)
Supplement: Supplementary file 1 — Supplementary Information 1. [file 41598_2023_38934_MOESM1_ESM.pdf]

Supplementary Figre S1 Location and ecosystem conservation status on Nakodo-jima. A red dot on the island represents a sampling point of soil used laboratory experiments in this study. (a) Degradation of grassland vegetation and erosion of surface soil (photo from 2001), (b) Eroded soil flowing into the inner bay year 2001), (c) Recovery of grassland vegetation after goat eradication (photo from 2015), (d) Continuous erosion of surface soils without vegetation recovery even after more than 15 years after goat eradication (year 2015). This blank map was modified from digital map provided by Geospatial Information Authority of Japan. (https://www.gsi.go.jp/ENGLISH/index.html)

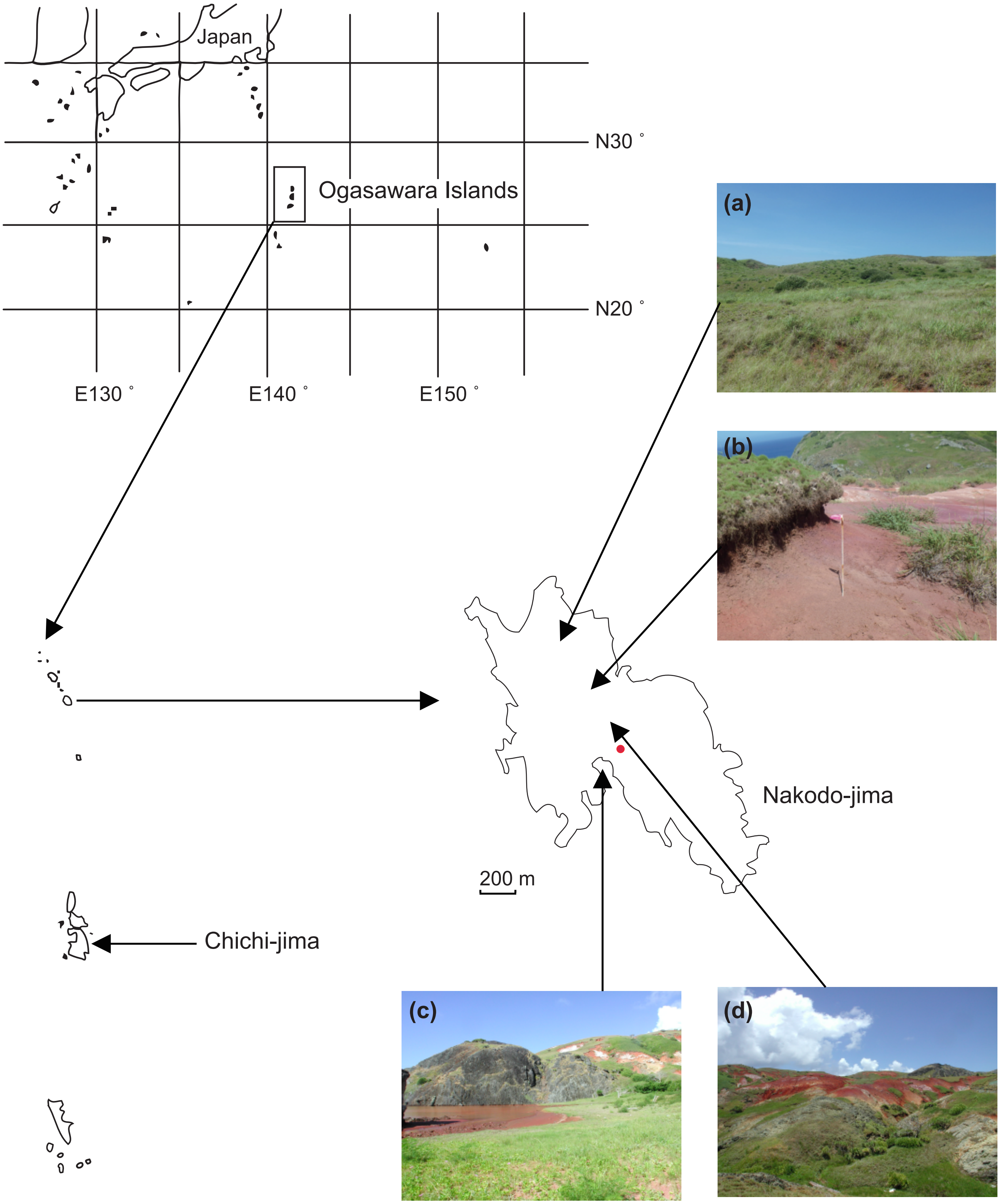

Supplementary Figre S2 Shoot:root ratios observed in 18 treatments. White, gray, and black bars represent the 0, 250, and 500 mg P_2O_5 kg soil^{-1} concentrations, respectively. Mean values (column) and standard errors (bar) are shown. (a) PasDil, *Paspalum dilatatum*; (b) PasScr, *Paspalum scrobiculatum*; (c) EmiSon, *Emilia sonchifolia*; (d) SpoDia, *Sporobolus diander*; (e) BidPil, *Bidens pilosa*; (f) SymSub, *Symphyotrichum subulatum*.

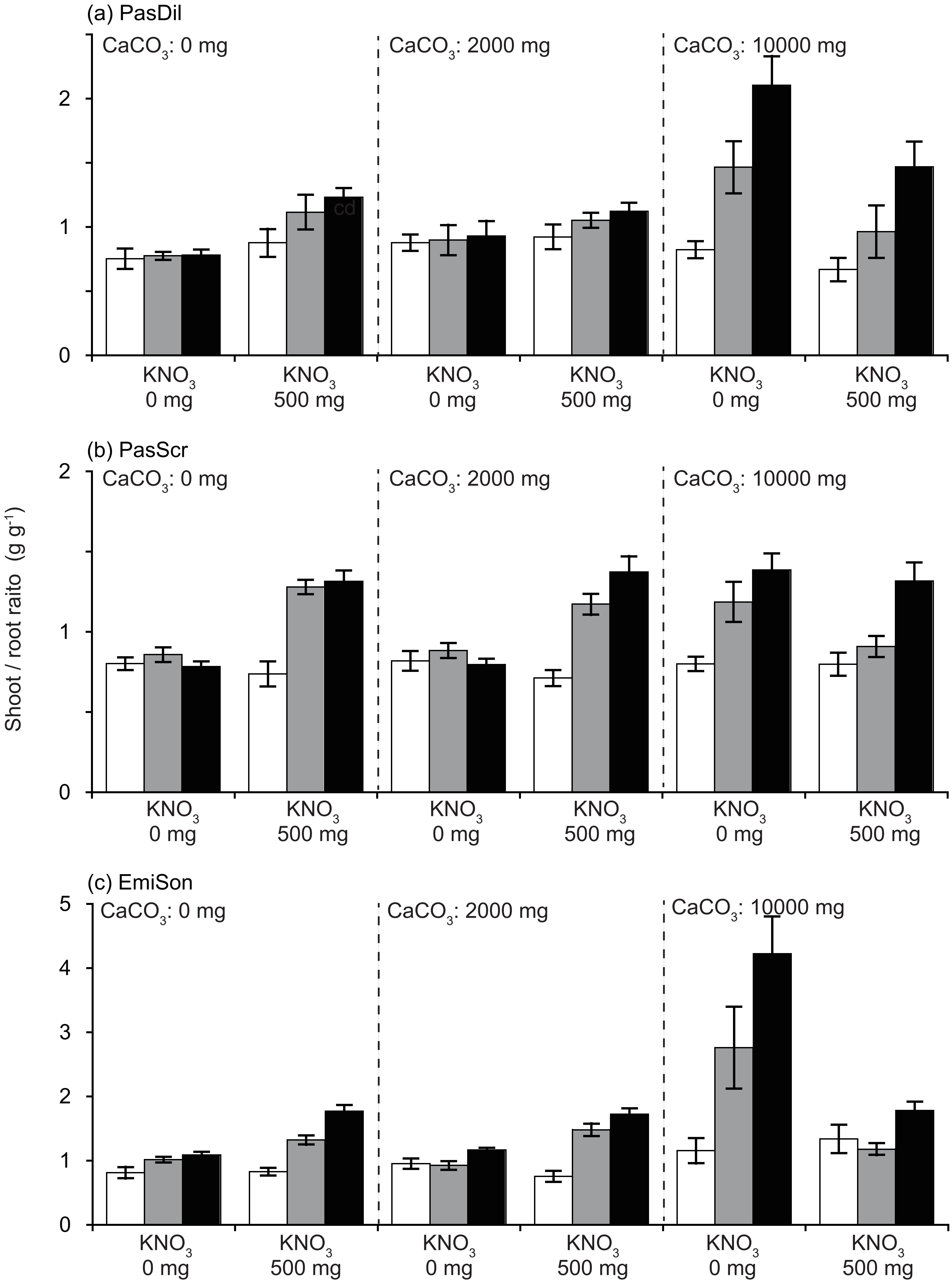

Supplementary Figre S2 Continued.

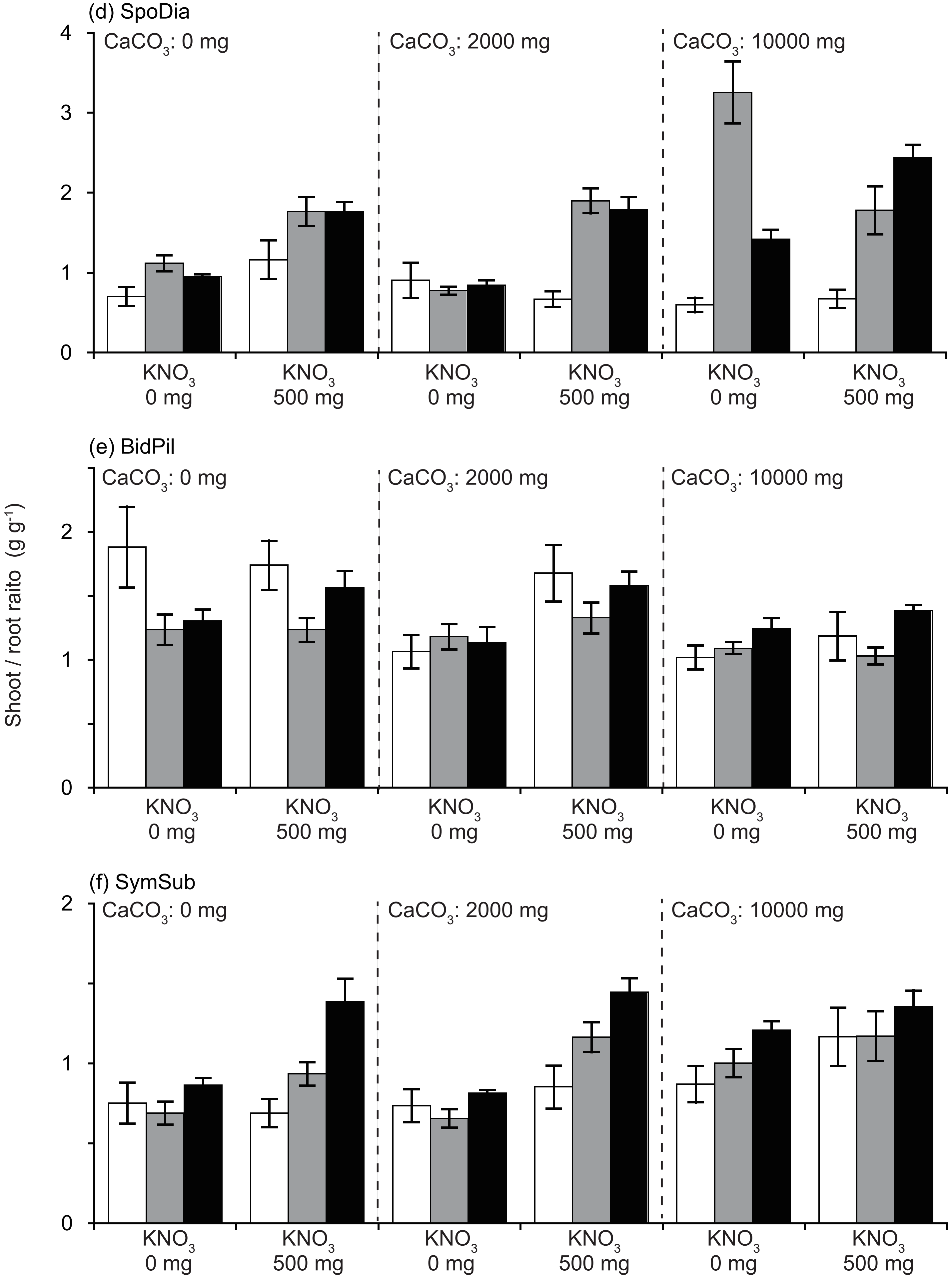

Supplementary Table S1 Life form, phenology, and seed morphologies of the six species.

Species	Life form	flowering season	Seed dispersal	Number of seed ($plant^{-1}$ yr^{-1})	Seed length (mm)
Paspalum dilatatum[1, 2]	Perennial	Jun. – Sep.	water, animal	25,000	2–3
Paspalum scrobiculatum[2]	Perennial	Aug. – Oct.	no data	no data	2–2.5
Sporobolus diander[3]	Perennial	May – Aug.	animal	no data	0.6–0.9
Bidens pilosa[1, 4]	Annual	Sep. – Nov.	wind, water, animal	3,000–6,000	4–16
Emilia sonchifolia[5]	Annual	Apr. – Nov.	wind	no data	3
Symphyotrichum subulatum[5]	Annual	Aug. – Oct.	wind	no data	2.5

References

[1] Weber, E. Invasive plant species of the waorld 2th edition. CABi, UK (2017)
[2] Osada T. Illustrated grasses of Japan. Heibonsha, Japan (1993) (in Japanese)
[3] http://www.worldfloraonline.org/taxon/wfo-0000901093 (view in March 8, 2023)
[4] Shimizu, T. Naturalized plants of Japan. Heibonsha, Japan (2003) (in Japanese)
[5] Satake, Y. et al. Wild flowers of Japan. Heibonsha, Japan (1991) (in Japanese)

Supplementary Table S2 Total amounts of carbon (C, %), nitrogen (N, %) and carbon/nitrogen ratio (C/N) of the subsoil collected from the Nakodojima. For the determination of total C and N contents, visible plant residues in the soil sample were carefully removed by using tweezers. The soil sample was thoroughly ground using a mortar and subjected to NC analysis (Sumigraph NC-22F, Sumika Chemical Analysis Service, Osaka, Japan).

C	N	C/N
0.84	0.07	11.4
